# The Morphology of the Dorsal Part of the First Rib in Neurogenic Thoracic Outlet Syndrome Patients: A Retrospective Clinical Study

**DOI:** 10.3390/jpm14020150

**Published:** 2024-01-29

**Authors:** Robert Fox, Franz Lassner, Andreas Prescher

**Affiliations:** 1 Institute of Molecular and Cellular Anatomy, RWTH Aachen University, 52074 Aachen, Germany; 2 Pauwelsklinik, Boxgraben 56, 52064 Aachen, Germany; flassner@gmail.com

**Keywords:** neurogenic thoracic outlet syndrome, nerve compression syndrome, costoclavicular exarticulation

## Abstract

Background: The recurrence or persistence of symptoms after thoracic outlet decompression (TOD) in patients with neurogenic thoracic outlet syndrome (NTOS) is not uncommon. Some authors have shown significantly better clinical outcomes in patients who underwent TOD with exarticulation of the first rib compared to a group who underwent TOD with preservation of the dorsal portion of the first rib. Several other case series have shown significant improvement after redo surgery with removal of the dorsal first rib remnant. This indicates the importance of the dorsal part of the first rib in NTOS. However, radical exarticulation may not always be necessary. In this study, we tried to answer the question of whether there is a morphological difference in the dorsal part of the first rib in NTOS patients that might help in the diagnosis and treatment of NTOS. Methods: We used the CT data of 21 NTOS patients who underwent TOD surgery and measured the dorsal part of the first rib, then compared them with a quota sample. Results: We found no difference in the dorsal part of the first rib between NTOS patients and the quota sample in our data. Conclusions: As there was no detectable difference, we were not able to use these data to help decide whether exarticulation is necessary in achieving adequate symptom relief. Therefore, we advocate exarticulation of the first rib when TOD is indicated.

## 1. Introduction

Thoracic outlet syndrome (TOS) is a rare type of compression syndrome. There are three different diagnoses depending on the precise structure that is compressed. In arterial TOS (ATOS), the subclavian artery is compressed in the scalene triangle. In venous TOS (VTOS), the subclavian vein is usually compressed between the clavicle and the first rib. In neurogenic TOS, there is compression of the brachial plexus. Neurovascular compression can occur at different anatomical levels, either the interscalene triangle, the costoclavicular space, or the pectoralis minor space [1,2].

Based on the actual diagnosis, the percentages of probable NTOS and VTOS cases appear to be approximately 80:20, with an incidence of NTOS between 2 and 3 cases per 100,000 people per year, with ATOS being present only in isolated cases [3]. The ratio of male to female patients was described by Leffert as approximately 1 to 3.5 [4]. Similar numbers supporting this approximation can be found in the demographic data of several clinical studies [5,6,7].

There has long been controversy surrounding NTOS and its exact etiology [8,9]. Trauma; anatomical variants such as fibrous ligaments, cervical ribs, anomalies of the first rib and pectoralis minor, and/or scalene muscle fibrosis; and hypertrophy or chronic spasms have been described as possible causes. Poor posture and repetitive arm movements (e.g., working on an assembly line) have also been linked to the development of NTOS [10,11,12].

Symptoms vary greatly. They include muscle weakness, sometimes with atrophy, numbness, paresthesia, and pain. The head, neck, shoulders, upper and lower arms, hands, and fingers can be affected. The symptoms persist or become worse when the patient exerts physical force on the arm, especially during overhead movements that stretch the brachial plexus [10,11,12].

The diagnosis of NTOS is difficult and complex because there is no specific, objective clinical test or imaging technique. It is currently a diagnosis of clinical exclusion, which, for many patients, results in years passing before they obtain a correct diagnosis and so often means a long journey of suffering [12,13].

In 2016, the North American Society for Vascular Surgery (SVS) established reporting standards for thoracic outlet syndrome. These standards state that three out of four of the following criteria must be present for a diagnosis of NTOS to be made [1]:

1. Local findings: Symptoms are consistent with irritation or inflammation in the area of compression, such as the scalene triangle. Complaints may be reported in the chest wall, axilla, neck, shoulder, upper back, or head. Palpation of the affected area will be painful.
2. Peripheral findings: Symptoms in the hand or arm consistent with compression of central nerves are noted. Symptoms include numbness, paresthesia, pain, and muscle weakness. Often these symptoms can be aggravated by specific maneuvers or reproduced by palpation of the affected region (e.g., scalene triangle).
3. Other diagnoses that could explain the complaints (cervical disc prolapse, orthopedic disorders of the shoulder, carpal tunnel syndrome, complex regional pain syndrome, brachial neuritis) have been excluded.
4. Positive test injection into the pectoralis minor muscle and/or scalene muscle.

Once NTOS has been diagnosed, the initial treatment for patients in Stages 1 and 2 (see Table 1) consists of physiotherapy, possibly combined with workstation modifications and activity restrictions for the affected arm. In our practice, it has proven to be effective to indicate surgical therapy in Stages 3 and 4. If surgery is not desired, conservative therapy with a trial injection of local anesthetic into the scalenus anterior and/or pectoralis minor muscle may be considered. Re-evaluation is usually performed after 4–12 weeks [14,15]. If the symptoms do not improve with conservative treatment, surgery is further recommended. The STOPNTOS Trial (Surgery Versus Continued Conservative Treatment for Neurogenic Thoracic Outlet Syndrome) has shown the superiority of surgery over continued conservative therapy in these cases [5].

In surgical decompression of the thoracic outlet, resection of the first rib plays a central role. One study in 2023 compared two groups of NTOS patients who underwent TOD [16]. One group underwent total resection of the first rib into the costovertebral joint; the other group underwent the conventional resection postulated by Roos, in which the posterior portion is left in place [16]. The patients from the exarticulation group showed significantly better results in the Disabilities of the Arm, Shoulder, and Hand Questionnaire (DASH) that was completed as part of the follow-up. However, as this study and others have also found good clinical outcomes with conventional surgery, it may not always be necessary to use this radical option [4,17]. As exarticulation of the first rib is associated with a higher risk of intraoperative complications, it would be desirable to identify patients in whom a less radical approach will result in adequate alleviation of symptoms.

Consequently, this raises the question of whether there are identifiable morphological differences in the dorsal portion of the first rib in NTOS patients compared to a normal population. The aim of this study was to investigate a potential typical, anatomical constellation which is pathognomonic for NTOS and if possible, to use the findings to guide diagnostic and therapeutic decisions.

## 2. Material and Methods

To address the question of the importance of the dorsal part of the first rib and define the distances to be measured, 180 pairs of rib bones were examined and measured, and the thoracic outlets of 14 cadavers from the Anatomical Institute of RWTH Aachen University were dissected. The data obtained were not otherwise used for this study.

For the manual radiological measurement of the first rib of the quota sample, a total of 100 CT thorax datasets were collected from the database of a supra-local radiological group practice with several branches. The first rib had to be scanned in its entirety and be of a good, assessable quality. For further inclusion and exclusion criteria, see Table 2.

The data were automatically anonymized by the program, so that no individual allocation and thus no reference to a specific person was possible. The data were further divided into age intervals of 5 years between 15 and 55 years, and the date of the CT scan was also removed to exclude a hypothetical allocation by age.

Patients’ CT data were obtained from 41 patients of the archives of a plastic surgery practice that had performed more than 400 operations on NTOS patients. Only CT data of patients with unilateral complaints, Grade 3 or 4 in the clinical classification of Table 2, who subsequently underwent unilateral surgery due to their complaints, were collected. The same inclusion and exclusion criteria applied to the patients’ image data as to the image data of the quota sample. CT data could only be obtained from female patients. Due to the aforementioned male/female ratio in NTOS patients, there were very few CT data samples from male patients, and all had to be removed after applying the inclusion and exclusion criteria.

**Figure 1:**
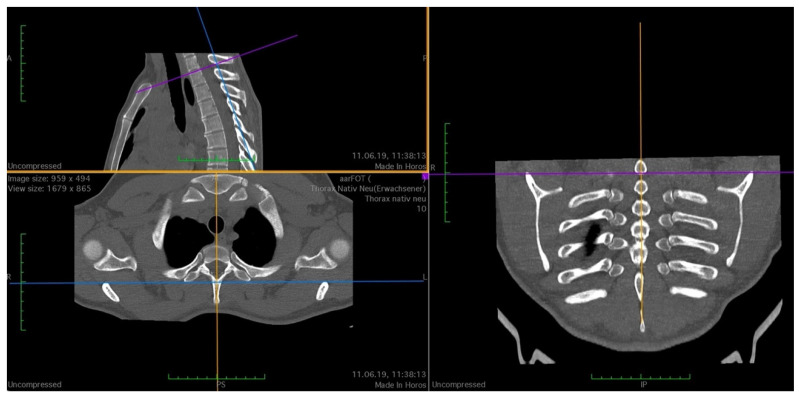
Setting the cutting planes in 3D MPR, purple line: horizontal plane at base plate of first thoracic vertebral body; yellow line: sagittal plane, aligned with the procc. spinosi of the thoracic spine; blue line: frontal plane, tangent to the processus transversi of the first thoracic vertebra.

To standardize the measurements, anatomical landmarks were defined to guide the orientation of the planes and the distances to be measured. As shown in Figure 1, the sagittal plane was aligned with the processus (procc.) spinosi of the thoracic spine, and the frontal plane was tangent to the processus transversi of the first thoracic vertebra. The horizontal plane was oriented as an axis of intersection through the two joints of the first rib or at the base plate of the first thoracic vertebral body, depending on the measurement point to be examined.

In close consultation with the lead surgeon, the distances (right and left) provided in Table 3 were defined as the points of measurement.

**Figure 2:**
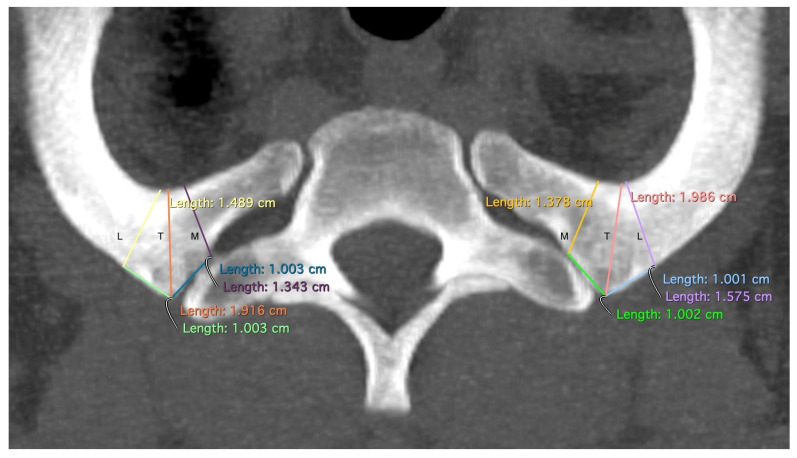
Measurement of T: the minimal width of the rib at the level of the tuberculum costae 1, M: the minimal width of the rib 1 cm medial to the tuberculum costae 1, and L: the minimal width of the rib 1 cm lateral to the tuberculum costae 1. Differently colored lines for better differentiation.

**Figure 3:**
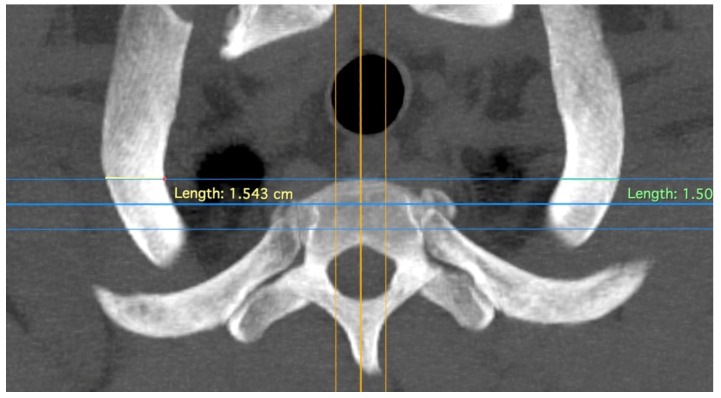
Measurement of rib width; thick yellow line indicating the sagittal plane; upper blue line marking the anterior edge of the first thoracic vertebra, the plane in which the Th1 root passes over the first rib with direct contact to the rib. Differently colored lines for better differentiation.

**Figure 4:**
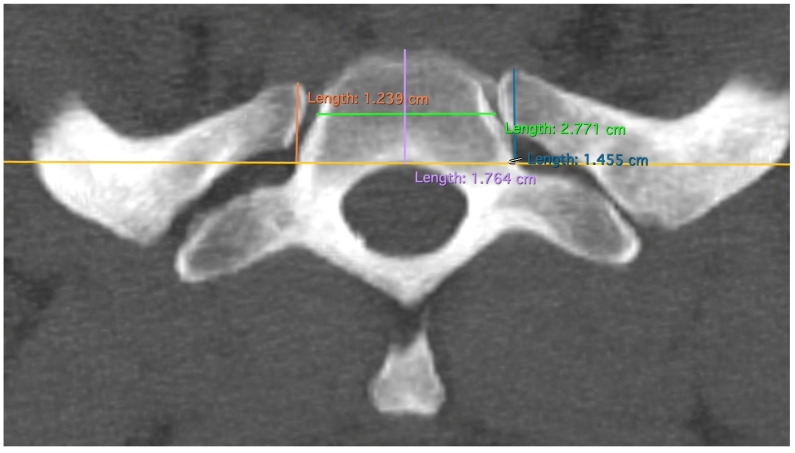
Measurement of RAP, VBW, and VBD; measurements of RAP as a perpendicular line to the tangent (yellow line) of the posterior edge of the first thoracic vertebral body. Differently colored lines for better differentiation.

The “T”, “M”, and “L” distances specifically measure the area of the first rib that remains when TOD surgery is performed conventionally and that may be responsible for recurrences (Figure 2). The point at which the width of the rib is measured—at the level of the anterior edge of the first thoracic vertebra—has been shown in our dissection of body donors to be the point at which the Th1 root passes over the first rib with direct contact to the rib (Figure 3). The RAP distance measures the proximal extension of the first rib and its relationship to the first thoracic vertebral body (Figure 4).

The depth and width of the first vertebral body were measured and statistically tested in the same way as the other measurements (Figure 4). The data from these two measurements were discarded in the interpretation because no conclusions could be drawn from them. Measurements were taken using Horos software (version 3.3.6). After multiplanar reformation (MPR) of the CT data, the rib could be displayed in sagittal, frontal, and horizontal planes and was now positioned and measured in a standardized manner.

**Figure 5:**
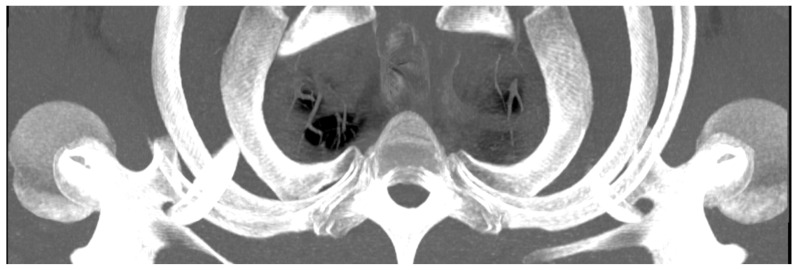
Example of significantly increased slab thickness showing multiple ribs in a caudocranial view.

The ‘slab thickness’—the thickness of the layer, shown in mm—was increased step by step, as not all structures of the area to be examined lie in one plane in a cross-section. This was performed so that all areas of interest were included in the image, but not covered by other structures. In this way, individual slices were superimposed on one another. This allows a thick cross-section of a three-dimensional object to be displayed in two dimensions, as shown in Figure 5.

All statistical analyses were performed using IBM SPSS Statistics version 28.0.

To ensure the reproducibility of the measurements and to exclude intra-rater bias, 15 randomly selected CT datasets were measured repeatedly. These included male and female subjects and patients with known NTOS. In total, the repeated measurements of 30 ribs and 15 vertebral bodies were compared.

Following the guidelines of Koo and Li, a reliability analysis was performed by calculating the intraclass correlation coefficient [18]. As this is an intra-rater reliability analysis, the model “twofold, mixed” and the type “absolute agreement” were selected. The CT data were then tested for a normal distribution. Although a normal distribution can be automatically assumed for a sample size >30, tests for normal distribution were performed to identify outliers and eliminate possible measurement or transmission errors [19]. To test for a normal distribution, we used the Shapiro–Wilk test and performed a visual analysis using quantile–quantile plots (Q-Q plots) [20]. The Shapiro–Wilk test for normal distribution showed that the values at one measurement point were not normally distributed. However, visually, a normal distribution could be assumed from the Q-Q plots.

Levene’s test was used to test the data for homogeneity of variance [21]. For the tubercle item, Levene’s test indicated homogeneity of variance; for all other items, the null hypothesis that there is homogeneity of variance between the three groups had to be rejected and the alternative hypothesis accepted.

The groups were then compared and tested for statistically significant differences. Means and standard deviations were calculated and a one-way analysis of variance (ANOVA) was performed. In order to avoid the problem of multiple testing, where alpha errors accumulate, post hoc tests were performed. The Scheffé test and Bonferroni correction were performed to analyze the homogeneity of variance; Dunnett T3 and the Games–Howell tests were performed to analyze the heterogeneity of variance. Post hoc tests were used with a significance value of 0.05.

Cohen’s d was calculated for the results to interpret the effect of significant mean differences.

Further comparisons were made using a visual statistical analysis to better interpret any differences in relevant measurements between the quota sample and the TOS patients.

## 3. Results

**Figure 6:**
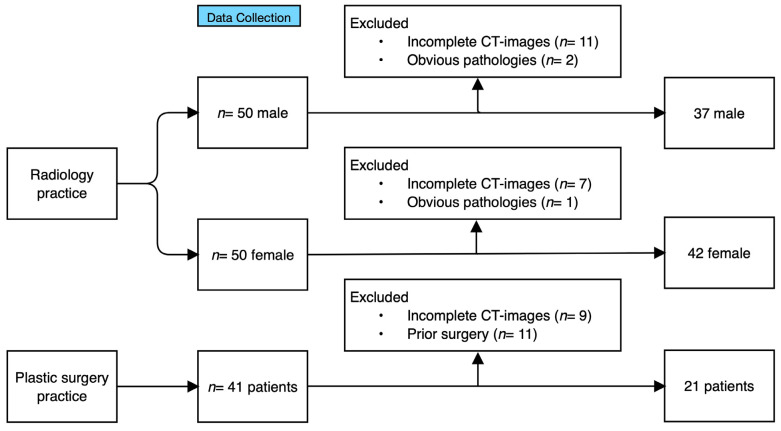
Flow chart showing the process of data collection and data processing.

On closer examination of the CT data in the quota sample than was possible at the time of data collection, and after applying the exclusion criteria, thirteen more CT records had to be removed from the male sample and eight more from the female sample (Figure 6). This left 37 CT scans in the male sample and 42 in the female sample. As CT imaging for the diagnosis of NTOS is controversial and represents a significant radiation exposure for the patient, CT scans are not regularly performed in NTOS patients [22]. After reviewing all available CT data and applying the inclusion and exclusion criteria, 21 patients’ CT scans remained to be processed. In the male sample, the age of the subjects ranged from 20 to 45 years (mean age interval: 31–35 ± 5 years). In the female sample, the age of the subjects ranged from 20 to 50 years (mean age interval: 36–40 ± 5 years). The TOS patients were all female and aged between 17 and 52 years (mean: 33.04 ± 11.04 years).

The results of the determination of the intraclass correlation coefficient showed “good” to “excellent” reliability for all measurement points, in the context of the definition of values provided in [18]. “Values less than 0.5 are indicative of poor reliability, values between 0.5 and 0.75 indicate moderate reliability, values between 0.75 and 0.9 indicate good reliability, and values greater than 0.90 indicate excellent reliability” [18].

Excellent reliability was achieved for six of the seven measurement points (“average measures” decisive). For two of the measurement points, the lower limit of the 95% confidence interval was “excellent”, for four measurement points, it was “good”, and for one measurement point, it was “moderate”. It is therefore unlikely that the results of this study are subject to intra-rater bias.

**Figure 7:**
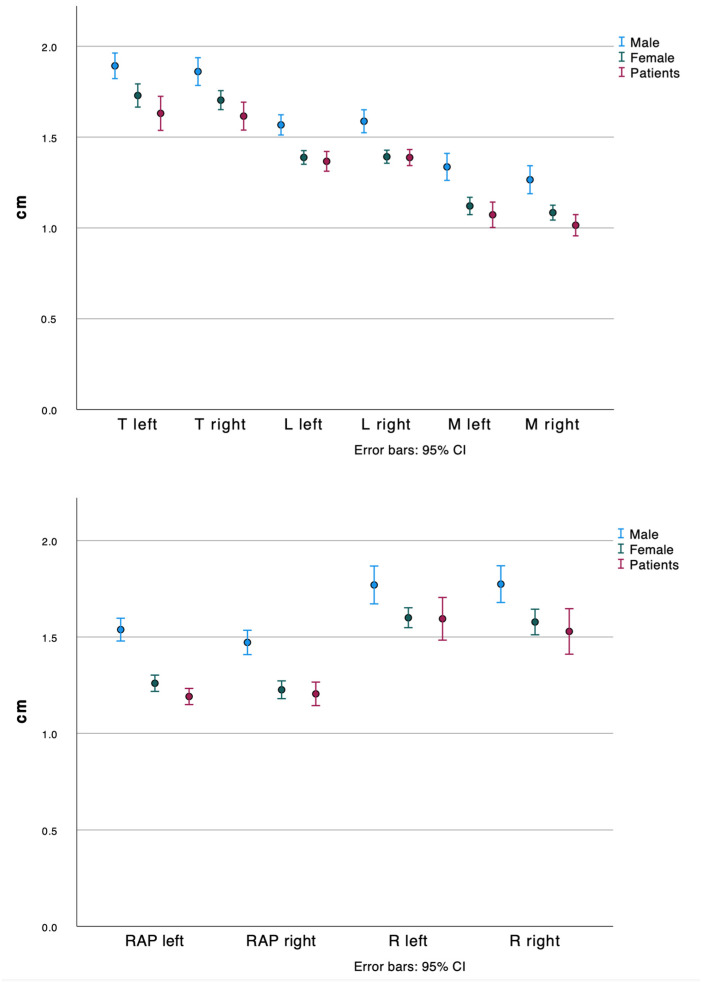
Two graphs, both showing grouped error bars of the mean values with 95% confidence interval (CI) of the distances measured (*x*-axis) in cm (*y*-axis). Overlap of red and green error bars indicating no significant difference between the patients (red, all female) and the female quota sample (green) in any measurement; no significant difference between the left and right side in any measurement in all groups; no overlap of the blue error bars with the green or red error bars indicating significant difference between the male (blue) quota sample and female quota sample as well as the all-female patients. T, tuberculum; L, lateral; M, medial; RAP, anterior to posterior rip extension; R, rib width.

The analysis of the mean differences and confidence intervals, as well as interpretation of the graphs (means with error bars), showed that there was a significant difference between the male and female quota samples for all measures (*p* < 0.05). There was also a significant difference between the male sample and the female TOS patients studied (*p* < 0.05). However, there was no significant difference between the female quota sample and the TOS patients studied at any of the measurement points (*p* > 0.05) (see Figure 7). There was also no difference between the right and left side in any of the groups (*p* > 0.05).

The results of the visual interpretation of the diagrams were confirmed by the results of the ANOVA with a simultaneous correction analysis.

As we found a significant difference between the male and female quota samples (*p* < 0.05), we determined Cohen’s effect size d for these differences. In order to interpret the mean differences, we followed the conventions suggested by Cohen, where d = 0.2 represents a small effect, d = 0.5 a medium effect, and d = 0.8 a large effect [23]. Thus, a medium to large effect was found for all measurement points.

## 4. Discussion

NTOS patients often have a long history of suffering and see an average of 4.7 doctors before a suspected diagnosis of TOS is made [14]. By the time surgical intervention is considered, they will have seen an average of 6.7 doctors [14]. However, after surgery, symptoms recur or persist in 5 to 30 percent of patients [7].

Postoperative scarring or an incomplete resection of the proximal segment of the first rib are the main reasons for recurrent and residual NTOS [7,24,25,26,27,28,29,30].

We were able to demonstrate significantly better clinical outcomes with the more radical approach of exarticulation of the first rib in a retrospective clinical study [16]. These findings are supported by further case series, where removal of the stump in patients with residual and recurrent NTOS after incomplete rib resection resulted in significant improvements [7,27,28,29,30].

Complete exarticulation of the first rib is technically demanding and time consuming [16]. In addition, it is not always necessary in all cases to achieve symptom-free patients [16]. However, at present, there are no preoperative imaging techniques or intraoperative indicators to help decide whether exarticulation is necessary.

The aim of this study was to discover any consistent morphological differences in the dorsal portion of the first rib that would enable tailored treatment to be provided to individual patients. 

Contrary to our expectation of finding wider ribs on the symptomatic side of NTOS patients, we found no detectable difference in the dorsal first ribs between the NTOS patients and women from a normal population.

There was also no difference found in the first ribs between the symptomatic side of the patients and the asymptomatic side of the patients. Therefore, it was not possible to use the data to develop a new tool to help diagnose or guide treatment.

Therefore, diagnosis will, for now, remain difficult and primarily clinical. This raises the question of a justifiable indication for a CT scan in TOS patients. We believe that—if there is no suspicion of VTOS or ATOS—a CT scan will not add value to the diagnosis and treatment decision in NTOS.

A CT scan would therefore do more harm than good in these mostly young patients. Such an examination is usually performed several times using different positions of the arm, and with a contrast medium, which increases DNA damage and in the area of radiation-sensitive organs such as the thyroid gland. As described in the reporting standards for thoracic outlet syndrome of the Society for Vascular Surgery, CT scans should only be used for specific clinical questions [1,31,32]. Bony abnormalities, such as the presence of a cervical rib, can be ruled out on a conventional anteroposterior X-ray of the thoracic outlet or via a chest X-ray.

However, this study has some limitations. Due to limited access to CT data of NTOS patients, we were not able to compare male NTOS patients with the male quota sample. Thus, we could also not address the question of why NTOS affects women significantly more often than men.

Our measurements focused on the width at different points of the most dorsal part of the first rib. However, the curvature of the first rib has not been taken into account. Sanders et al. discussed the use of the first rib’s curvature in a chest X-ray to guide the decision whether first rib resection should be considered at all, or if scalenectomy alone might be enough [33,34,35]. 

**Figure 8:**
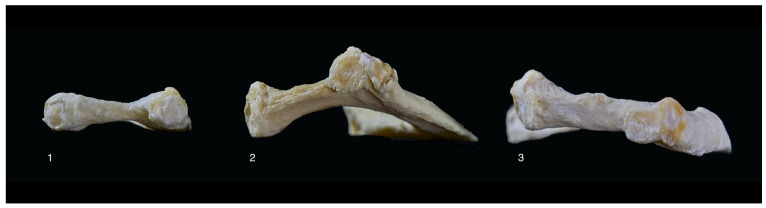
Three first rib bones numbered 1, 2, and 3 demonstrating different variations in a posterior to anterior view from left to right.

A further limitation is that our measurements were taken only in the horizontal plane. In our investigation of over 180 first rib bones, we have also seen a variation in the curvature in the vertical plane, as shown in Figure 8. Detectable especially when put on a flat surface, the rib might be completely flat (Figure 8, rib 1), angled upwards (Figure 8, rib 2), or angled downwards (Figure 8, rib 3).

This could play a role in the hypothesis that cranialization of the plexus by the first rib leads to an increased tension of the root Th1 and might be exacerbated by a first rib that is angled upwards [16]. Further study is needed and might need more advanced techniques like the comparison of three-dimensional models to provide accurate results.

Although a redo procedure can be performed without any significant morbidity, as described by Likes et al. (2014), there is still a considerable risk to redo surgery that cannot be neglected [27,30]. Due to postoperative scarring—especially of the long thoracic nerve—the dorsal scapular nerve, the phrenic nerve, and the subclavian artery are clearly endangered.

In centers where both a transaxillary (TA) and supraclavicular (SC) approach are performed, using TA-TOD as the primary approach and SC-TOD in a redo scenario might be a way of reducing the risk [7].

Since no directional difference could be shown, we believe that it is advisable to choose the radical approach with exarticulation of the first rib for patients in whom surgical resection of the first rib was indicated to reduce the number of possible recurrences of TOS symptoms.

## Figures and Tables

**Table 1:** Thoracic outlet syndrome classification [16].

Stage	Symptoms
0	None, no limitations for physical load.
1	Symptoms when submitted to a severe physical load, with corresponding occupational limitations; symptom-free in daily activities.
2	Symptoms when submitted to a moderate physical load, with limitations in daily activities.
3	Symptoms under a light physical load; severe limitations in daily activities.
4	Permanent symptoms; motor and sensory deficits.

**Table 2:** Inclusion and exclusion criteria of CT data.

Inclusion criteria	Age between 17 and 55. Complete representation of the first rib in the CT thorax image. Good image quality (slice thickness 1.5 mm). Stage 3 or 4 (see Table 1, NTOS patients only).
Exclusion criteria	Severe deformity of the spine (e.g., scoliosis) or thorax. Deformities of the first rib (e.g., after trauma). Foreign bodies affecting the image (e.g., pacemaker). Previous surgery on the first rib or vertebral body Th1 visible in the picture. Obvious pathological changes (e.g., fractures, tumors, osteophytes).

**Table 3:** Description of the rib measurements.

Variable Name	Abbreviation	Description
Tuberculum	T	The minimal width of the rib at the level of the tuberculum costae 1
Medial	M	The minimal width of the rib 1 cm medial to the tuberculum costae 1
Lateral	L	The minimal width of the rib 1 cm lateral to the tuberculum costae 1
Rib width	R	Minimal width of the rib at the height of the front edge of the first thoracic vertebral body.
Anterior to posterior rip extension	RAP	From the most ventral point of the caput costae 1, a perpendicular line to the tangent of the posterior edge of the first thoracic vertebral body.
Vertebral body width	VBW	Width of the first vertebral body at the level of the centre of the articulatio capitis costae 1
Vertebral body depth	VBD	From anterior edge to posterior edge (most ventral point of vertebral foramen) of the first vertebral body

## Data Availability

Data available on request. The data presented in this study are available on request from the corresponding author. The data are not publicly available.
